# A Therapeutical Arrangement of the Materia Medica; or the Materia Medica Arranged upon Physiological Principles, and in the Order of the General Practical Value Which Remedial Agents Hold under Their Several Denominations, and in Conformity with the Physiological Doctrines Set Forth in the Medical and Physiological Commentaries

**Published:** 1843-04

**Authors:** 


Art. II.—*A Therapeutical arrangement of the Materia Medica; or the Materia Medica arranged upon Physiological Principles, and in the order of the general practical value which remedial agents hold under their several denominations, and in conformity with the Physiological doctrines set forth in the Medical and Physiological Commentaries.* By Martyn Paine, M. D., A. M., author of the Commentaries, and of the Letters on the *Cholera Asphyxia* of New York, and Professor of the Institutes of Medicine and Materia Medica in the University of New York. New York: J. &. H. G. Langley, 57 Chatham Street, 1842, p. 271 duodecimo.

This little volume presents as fair a claim to the motto, "*multum in parvo*," as any production of the American press, or indeed of any press, that has fallen under our notice. And it contains, for its size, not only a *large amount* of matter, but matter of a practical and valuable character. Were we inclined to appear, on the occasion, either classical or poetical, or both at once, we might correctly enough apply to it the distich of the great and accomplished Mantuan, in his poem on bees;

"In tenui labor; at tenuis non gloria, si quem
Numina læva sinunt auditque vocatus Apollo."

The title-page itself of the work so far discloses its character and design, that our readers need but little more for the attainment of a competent knowledge of them. From that page they will at once perceive, that Professor Paine is a thorough-bred and uncompromising vitalist. Hence they cannot fail also to learn from it that he considers the science of medicine as consisting in a true and complete exposition of the laws of living organized matter; and the practice of medicine in a correct application of those laws to the treatment of diseases—the means of treating them being derived from Materia Medica. And they will further be made sensible, that, as a writer and teacher, he denies the existence of any identity, connexion, or even close analogy between the philosophy of living and the philosophy of dead matter. In more express and definite terms, they will thus learn, that he regards physiology, in its principles and laws and their mode of action, as essentially different from those of either chemistry or the science of mechanics; and as being an exposition of functions exclusively *vital.*

Still however, though so much, respecting the character and design of the volume may be ascertained from the title-page, we think it best to *lay* before our readers a brief extract from its preface, in more definite explanation of them.

"These purposes" (those of the work we are considering) are mainly:

"1. To arrange the *Materia Medica* upon intelligible, physiological, and therapeutical principles.

"2. To indicate the relative therapeutic value of the various articles, under their different denominations, by arranging them in the order of their value.

"3. To give to the student a comprehensive and ready view of the merits of the various articles composing the *Materia Medica*, and of their relations to each other *physiologically.*

"4. To supply a convenient means of graduating the doses of medicine."

From that portion of the work which is headed "General principles," we shall also make a brief extract, our object in doing which will presently appear.

"1. All remedies operate upon the same principle as *morbific agents*, and all become morbific when injudiciously applied. Applied to healthy systems, they alter the vital properties and actions so as to constitute disease. * * * "Medicines" says Linnæus, "differ from poisons, not in their nature, but in their doses." And so Pliny; "*ubi virus, ibi virtus.*"

"3. All curative agents operate upon the *morbid properties*, either directly or indirectly through sympathy, and produce their salutary results, by so altering the morbid properties, as to enable them to take on their natural tendency to a state of health."

These three last quoted paragraphs show our author to be a liberal, bold, and independent thinker; and we fully concur with him in the sentiments they express. *Medicine* and *poison* are but different names for the same article, according to the quantities and modes *in* which, and the circumstances *under* which it is administered. And all remedies which actually change disease are necessarily *alteratives*, from the nature of the case, and the meaning of the term.

As relates however to the third paragraph, our coincidence in opinion with the Professor is not quite so entire. At least we do not like the expression "operate on properties" whether they are morbid or healthy; because we think it calculated to make an erroneous impression. Agents do not act immediately on "properties," which have no existence apart from the substances to which they belong. They are but the attributes, signs, or shadows of such substances. To operate on *them* therefore is tantamount to operating on a non-entity. The operation is *on* the substance and the change produced is *in* the substance; and the properties of course experience also a corresponding change; because as is the substance, so necessarily are they. This is true of all properties, whether the substances they belong to be living matter or dead matter.

In illustration and proof of this, let us consider for a moment the properties of elasticity and weight. We cannot operate *immediately* on them. To change them, we must change the substances to which they belong. The production of an alteration in properties then is of necessity an indirect process. The reason is plain. It must reach them through the substances to which they appertain.

We should not have noticed this error in *mere words*, had they been the words of an ordinary writer; or had they been employed but once even by Professor Paine. In either case we might have regarded them as slips of the pen, and allowed them to pass without animadversion. But the Professor is able in intellect, and distinguished and influential as a writer and teacher, and has repeatedly used them in the course of his work. His errors therefore, whether in words or thoughts, are of bad example, and dangerous, because they lead weaker minds astray. Hence their rectification is necessary and important.

Professor Paine arranges the articles of Materia Medica under twelve heads, which he denominates and classes in the following order, and under the names attached to them.

"1. Antiphlogistics. 2. Permanent Tonics. 3. Diffusible Stimulants. 4. Cerebro-Spinants, or Nervous Agents. 5. Astringents. 6. Uterine Agents. 7. Urinary Agents. 8. Anthelmintics. 9. Errhines. 10. Chemical Agents. 11. Diet and Regimen, in a general sense."

This arrangement (and we presume not to say that it can well be improved) does not amount to a complete execution of our author's plan, already communicated in his own words. It does not we mean, in every particular, strictly conform to physiological requirement. This is the case with the class of

"Tonics," that term not being properly applicable to *living* matter in any condition it can be made to assume, but to dead matter in a state of *tension.* This may be illustrated by the condition of well prepared bows, and by that of drums, harps, violins, and other instruments of music. These several instruments must be in a "tonic," that is a *tense* condition, when well fitted for the purposes for which they are designed—the first to lanch the arrow, and the others to send forth well-conditioned musical sounds. As regards the condition of the bow and bow-string, the cords of the harp and violin, and the head of the well-braced drum, nothing at all analogous to it is produced by the action of "Tonics" on any portion of the human body. In the condition of a healthy and vigorous muscle, nerve or gland, there is no more of *tone* or *tension,* in the true sense of the terms, than there is in the condition of a deranged nerve or muscle. The word *tone* belongs to *mechanical,* not to *vital* philosophy. Still, however, provided we divest it entirely of its literal, mechanical, and coarse meaning, and so interpret it as to signify soundness and excellency of condition, it *may,* without communicating such mechanical views as are highly injurious, be applied to vital matter. And we confess our inability to substitute for it a better term.

We fully however concur with our author, that what are called "*tonic*" remedies are greatly overrated, as relates to their usefulness. In many cases, especially of chronic diseases, they *may,* and we doubt not *do, as alteratives,* prove serviceable. But, from the injudicious manner, in which they are exhibited, we have long been convinced that, in convalescence from *acute* disease, they do an immensity of mischief. Indeed, in such cases, provided the complaint be sufficiently deracinated, by antecedent means, they are wholly unnecessary; and usually injurious. When, by a judicious treatment, an acute disease is brought down to the proper point, *nature,* by her recuperative energies, most safely and effectually completes the cure. The best tonic under those circumstances is a well directed regimen and form of diet. Every thing beyond that embarrasses nature, and impedes her in her efforts to remove the complaint. Nor is this view of the matter at all inconsistent with the fact, that tonics are efficaciously employed in the treatment of intermitting and remitting fever. That, even in those complaints however, tonics, unskilfully administered, are productive of much mischief, is a circumstance familiar to every experienced practitioner of medicine.

To the class-term "Astringents" similar remarks are correctly applicable. It is not, in its import, strictly physiological. True; the articles thus denominated produce an astrictive effect on the lips, tongue, and fauces of those who swallow them. But there the astriction stops. Though the functions of the system may be affected by the article, the affection is alterative, not astrictive.

To the class-term "Anthelmintics" also exception may be taken on the same ground. It bears no direct relation to physiology—certainly none to the physiology of man, whatever may be its relation to that of other animals. It signifies articles *deletereous to worms*, and nothing more. Of the effect of those articles on the human system it is in no way expressive.

Let it be distinctly understood however, that these animadversions on some of his terms are not to be construed into a sentiment of disapproval toward Professor Paine, as a writer. Far from it. We do not remember any terms more suitable that might be offered as substitutes for those to which we have excepted. The defect in the class-names arises much more from the intractable nature of the case than from any fault in our author. The result is therefore matter of *regret*, because it *cannot* be amended, rather than of *blame*, because it *is not*.

With the Professor, in his views respecting "Cold" we cannot concur. He arranges and treats the article as a *direct sedative*. We, on the contrary, regard it as a *direct stimulant* and an *indirect* sedative. To the *nerves* it is not only a direct, but also a very *powerful* stimulant. It may be made to produce keen and intensely painful sensations—a result which cannot possibly be the product of a sedative.

Nor is cold a stimulant to the nervous tissue alone. It also acts most powerfully as such on the vascular system. When carried to a sufficient extent, it not only burns, but actually *blisters*. A piece of frozen quicksilver received into the hand burns and blisters like red-hot iron—an effect totally foreign from the influence of a sedative.

Frozen quicksilver and red-hot iron however, although they produce the same effect on the hand, do so in modes the very reverse of each other. The iron burns and blisters by pouring caloric superabundantly *into* the hand; the quicksilver by drawing caloric superabundantly *out of it*. In doing this it brings the caloric, in a large amount, to a *focal point,* in consequence of which it acts like the solar rays, brought to a point, by a powerful burning lens.

Such are our views respecting the mode of operatiou of frozen quicksilver. And whether they are right or wrong, the fact they relate to is certain. The quicksilver which is intensely cold, excites a blister. But this it could not do, were it not a stimulant.

Cold is an antiphlogistic remedy, because it is an *evacuant.* And, so far as they are evacuants, all medicinal substances are antiphlogistics—as necessarily and essentially so, as blood-letting itself—though not in an equal degree.

Blood-letting alone excepted, all evacuations from the body are the offspring of secretion. Except the fæcal discharge, evacuation is but the discharge of secreted matter.

If this be true (as it unquestionably is) secretion is nature's chief mode of removing inflammatory affections. Thus the secretion and evacuation of bile cure hepatitis. The secretion and discharge of mucus aid in the removal of the several kinds of thoracic and pulmonary inflammation. A superabundant secretion and evacuation of saliva take down inflammation of the salivary glands. A copious secretion and discharge of urine aid in the cure of nephritis. The secretion and evacuation of pus contribute to the cure of the inflammation and congestion attendant on abscesses. And the secretion and discharge of perspirable matter aid in the removal of every affection to which man is subject. To the cure of all *febrile* affections they are indispensable.

From these considerations we have long been of opinion that there is just ground for the formation of an *order* at least, if not a *class* of remedies, under the denomination of SECRETORIES. And it would be an extensive and important one. For we venture to say that every remedy, which contributes to the cure of a general disease, does so, to no small extent, by throwing into natural and healthy action some of the secreting organs of the body. Hence arises what is denominated, by most of the ancient medical writers, and by not a few of the modern ones, the *critical discharge.* And though the regular occurrence of such discharge is even tauntingly discredited, by many physicians of the present day, it is notwithstanding true. The evacuation comes most frequently from the liver and alimentary canal, the mucous lining of the trachea and lungs, the kidneys, or the *skin.* But no matter whence it comes. Its existence is certain. And when of a local character, as it often is, it proceeds from the organ, or its neighborhood, where the deepest congestion exists; and it relieves or removes that congestion.

The critical discharge is the product of nature; not of medicine. Nor does the good it effects arise from, or depend on, either its quantity, its kind, or its quality—nor from all these attributes united. It is to be regarded but as indicative of good, as evidence we mean that the course of action in the system of the sick is changed. And that change of direction is from *centripetal* to *centrifugal*—a change which is always salutary and of good promise. In febrile complaints the course of action is from without to within; and usually secretion of every description is restricted. And when the action changes its direction, from within to without, the secreting organs are thrown into operation again; and the result of that change is the restoration of health. But we can pursue this desultory notice no farther. In a few general and brief remarks, therefore, we shall bring it to a close.

The volume we have been considering, though small, exhibits notwithstanding a large amount of acquired knowledge, and no small degree of strong, independent, and well matured thought. Its arrangement, and the peculiar tenor of its doctrines and directions, are entitled to our entire and decided approval. We regard it, however, as little else than a syllabus or text-book for Professor Paine's course of lectures on Materia Medica. And, as such, we deem it peculiarly valuable to the pupils to whom those lectures are delivered. To them, during their pupilage, it will prove a highly convenient and useful companion in the Professor's class-room, as well as in their own studies, and even when subsequently engaged in professional practice, a ready and abundant remembrancer of what they have already learned. Nor, limited as the work *is* on every topic, will even those, who have not attended the lectures of its author, fail to derive from a careful perusal of it many important and some new philosophical views of Materia Medica. In these respects, therefore, the volume is cordially recommended to public attention.

